# Assisted Reproduction in Congenital Adrenal Hyperplasia

**DOI:** 10.3389/fendo.2019.00723

**Published:** 2019-10-23

**Authors:** Anastasios Chatziaggelou, Evangelos G. Sakkas, Raffaella Votino, Maria Papagianni, George Mastorakos

**Affiliations:** ^1^Department of Obstetrics and Gynecology, General Hospital of Drama, Drama, Greece; ^2^Unit of Endocrinology, Diabetes Mellitus and Metabolism, 2nd Department of Obstetrics and Gynecology, Medical School, Aretaieion Hospital, National and Kapodistrian University of Athens, Athens, Greece; ^3^Pediatric Endocrinology Unit, 3rd Department of Pediatrics, Medical School, Hippokrateion General Hospital of Thessaloniki, Aristotle University of Thessaloniki, Thessaloniki, Greece

**Keywords:** infertility, IVF (*in vitro* fertilization), congenital adrenal hyperplasia (CAH), pregnancy, assisted reproduction (ART)

## Abstract

Congenital Adrenal Hyperplasia (CAH) is a group of autosomal recessive disorders characterized by defects of adrenal steroidogenesis due to mutations in one of the following enzymes: 21-hydroxylase (21OH), 11β-hydroxylase (11βOH), 17α-hydroxylase (17OH; also known as 17, 20-lyase), 3β hydroxysteroid dehydrogenase type 2 (3βHSD2), steroidogenic acute regulatory protein (StAR), P450 cholesterol side-chain cleavage (P450scc), and P450 oxidoreductase (POR). More than 95% of congenital adrenal hyperplasia cases are due to mutations in CYP21A2, the gene encoding the adrenal steroid 21-hydroxylase enzyme (P450c21). This work focuses on this type of CAH given that it is the most frequent one. This disease is characterized by impaired cortisol and aldosterone production as well as androgen excess. A variant of the CAH is the non-classic type of CAH (NCCAH), usually asymptomatic before the 5th year of age, diagnosed during puberty especially in patients visiting a fertility clinic. NCCAH is characterized mainly by anovulatory cycles and/or high androgen concentrations. Both types of CAH are associated with infertility. Given that the incidence of NCCAH is greater than that of CAH, patients suffering from NCCAH are more often diagnosed for the first time in a fertility clinic. Thus, screening for NCCAH should always be considered. The causes of infertility in CAH patients are multi-factorial including virilization of external genitalia, altered psychosocial development, and hormonal disorders. The main challenges encountered in assisted reproduction are the androgen excess-associated anovulatory cycles as well as the increased circulating progesterone concentrations during the follicular phase which impact endometrial receptivity, tubal motility, and cervical thickness. Administration of sufficient substitution dose of glucocorticoids usually resolves these problems and leads not only to successful assisted reproduction treatment but also to spontaneous pregnancy. Patients with CAH should be followed by a multidisciplinary team including gynecologist, endocrinologist, and pediatrician.

## Introduction

Congenital adrenal hyperplasia (CAH) is a group of seven autosomal recessive diseases. The genes responsible for congenital adrenal hyperplasia encode enzymes involved in cortisol biosynthesis. These enzymes are: 21-hydroxylase (21OH), 11β-hydroxylase (11βOH), 17α-hydroxylase (17OH; also known as 17, 20-lyase), 3β hydroxysteroid dehydrogenase type 2 (3βHSD2), steroidogenic acute regulatory protein (StAR), P450 cholesterol side-chain cleavage (P450scc), and P450 oxidoreductase (POR). Multiple hormonal imbalances occur and CAH manifests with a range of clinical and biochemical phenotypes, with or without alterations in glucocorticoid, mineralocorticoid, and sex steroid production. Congenital adrenal hyperplasia can be distinguished clinically in two forms, “classic” and “non-classic” (non-classic CAH; NCCAH) (1). Mutations in these enzymes result in reduced cortisol production, which leads in its turn to increased secretion of corticotrophin releasing hormone (CRH) and adrenocorticotropic hormone (ACTH) causing adrenal cortex hyperplasia (2). As a result, precursor steroids accumulate before the point of enzymatic disruption shifting the biosynthetic pathway toward production of sex steroid hormones, more specifically adrenal androgens, which are found in excess. Approximately 90–95% of cases with CAH are attributed to 21OH deficiency (3). Clinical distinction to “classic” and “non-classic” forms depends on the severity of the clinical expression of this deficiency (3). “Classic” CAH can also be distinguished in two forms, “salt-wasting” and “simple virilizing.” Seventy-five percent of cases with “classic” CAH represent the “salt-wasting” form presenting with cortisol and aldosterone deficiency. In “salt-wasting” CAH, the enzyme activity is completely silenced. In “simple virilizing” CAH, there is 1–2% enzyme activity with normal mineralocorticoid concentrations. In NCCAH, enzyme activity is satisfactory (20–50%) and thus, patients remain asymptomatic, or symptoms appear much later (this form is otherwise called “late-onset CAH”) (3). Females with NCCAH are not virilized at birth (4). The incidence of “classic” CAH is 1:10000–1:20000 live births and that of NCCAH 1:1000 live births (3, 5). This classification is artificial because CAH has a wide and continuous range of clinical features depending on residual enzyme function (5).

Pregnancy rate is related to the clinical severity of the disease (6, 7). Severe infertility is associated with “salt-wasting” CAH. The low rates of fertility in women with “classic” CAH are also related to the decreased *libido* of these patients, with less chance of heterosexual relations and less desire to engage in family formation (8, 9). Many women with NCCAH present with mild symptoms and therefore remain undiagnosed. It is difficult to assess accurate infertility rates in NCCAH (8).

## Fertility in CAH

### Fertility in “Classic” CAH

Fertility rates in women with “classic” CAH and especially those with “salt-wasting” CAH are significantly lower compared to those in the general population (6, 9–12). On the other hand, it is difficult to estimate these rates in women suffering from CAH because they do not usually seek pregnancy while studies include a small number of patients. These rates are improved when studies include only women with CAH who are actively trying to conceive which means women who undergo surgical and/or pharmaceutical therapy (8, 10). Indeed, in a study of 81 women with “salt-wasting” CAH evaluated since birth, only nine sought pregnancy, with eight of them conceiving in the end (8, 13). Independently of these women's infertility, pregnancies are usually normal and uneventful (11).

### Fertility in NCCAH

“Non-classic” CAH is a frequent cause of infertility, often undiagnosed (14). Pregnancy rates in women with NCCAH who visit infertility clinics due to infertility or hyperandrogenemia, vary according to studies and range between 65 and 95% (8, 15–18). There is a significant phenotypic overlap between PCOS and NCCAH, often leading to misdiagnosis of patients who seek advice in fertility clinic. Patients with PCOS manifest hirsutism, hyperandrogenemia, variable degrees of insulin resistance, and anovulation (19). Because of the similarity of clinical features between the two conditions, it has been postulated that about 33% of patients diagnosed with PCOS actually suffer from NCCAH (20). Due to the different treatment of these two conditions and the possible incidence of NCCAH in the fetus during pregnancy, the correct differential diagnosis of patients with infertility is critical (20).

### Etiology of Infertility in CAH

The etiology of infertility in patients with CAH is multifactorial, including ambiguous genitalia and their complications, excessive androgen secretion, adrenal progesterone hypersecretion, co-existence of PCOS, and various psychosocial factors (5–7, 11, 14, 20–22).

Female fetuses with “classic” CAH and adrenal androgen hypersecretion during endometrial life present with malformations of external genital organs such as presence of a urogenital sinus, labial fusion, and variable degrees of clitoral hypertrophy. These malformations render sexual intercourse unpleasant and sometimes prohibitive, reducing the chance of pregnancy. The possibility of sexual intercourse is related to introital width, vaginal length, and clitoral integrity. Internal genitalia remain intact (8). Exposure to increased androgens in endometrial life also affects psychosocial development of these patients. Women with “classic” CAH, experience gender-related disorders such as participation in games of masculine orientation as children and pursuit for men's occupations in adult life. An increased rate of homosexual and bisexual relationships among patients with CAH is reported. In addition, reduced *libido* and decreased desire for family formation are observed (8, 9, 20).

Chronic exposure to adrenal androgens causes disorders of the hypothalamic-pituitary-ovarian axis leading to hypersecretion of LH. In addition to increased androgen concentrations in peripheral blood, CAH is also associated with increased concentrations of progesterone. Progesterone secretion in these patients is continuous resulting to modification of GnRH pulsatility, prevention of normal endometrial development, defective quality of cervical mucus, and decrease of tubal motility, resulting thus, in significant decrease in fertility (7, 8, 14, 20). Thirty to sixty-eight percent of women with “salt-wasting” CAH and 30–75% of women with “simple-virilizing” form manifest menstrual irregularities and anovulation (10).

## Management of Infertility in Women With CAH

### Treatment of CAH

Women with “classic” CAH have ambiguous external genitalia. Female embryos are exposed to adrenal androgens from the 7th week of pregnancy resulting in clitoral enlargement, fusion, and scrotalization of the labial folds, and rostral position of the urethral/vaginal perineal orifice, placing the phallus in male position while the internal female reproductive organs are developing normally. These changes are classified according to the five Prader stages (14). In case of patients with “classic” CAH and ambiguous genitalia, the possibility of surgical rehabilitation should be considered (23). It includes clitoroplasty, vaginoplasty, and labiaplasty and aims at removing redundant erectile tissue, preserving the sexually sensitive gland clitoris, and providing a normal vaginal orifice that functions adequately for menstruation, intromission, and birthing. In addition these interventions protect from recurrent urinary tract infections which result from pooling of urine in the vagina or urogenital sinus (10, 14, 23, 24). The main complications of surgical interventions include urinary incontinence, clitoral pain, painful intercourse and inadequate introitus, vaginal stenosis, and anorgasmia. These complications lead to decreased intercourse frequency. Secondarily, they have been observed strictures, fibrosis and scarring, fistulas, and recurrent urinary infections (10, 14, 24). Glucocorticoid therapy is the pharmaceutical treatment of choice, both for patients with “classic” CAH and for those with NCCAH, with addition of mineralocorticoid to patients with “salt-wasting” CAH (9α-fludrohydrocortisone acetate) (1–3, 9, 23). Glucocorticoids substitute the deficient endogenous cortisol synthesis and thus, CRH and ACTH hypersecretion is reduced, leading to decreased adrenal androgens secretion (25). Subsequently, progesterone levels are reduced and normal ovulation, endometrial proliferation and implantation ensue (5, 12). In “classic” CAH, to control overnight HPA-driven increase of adrenal androgens, a variety of glucocorticoid treatment regimens have been used. Treatment with hydrocortisone administration in three equal doses (starting at 8.00 am) seems to be the most appropriate. Many specialists used to administer prednisolone in adult patients because of the more convenient dosage regimen. However, this treatment is gradually abandoned because it is accompanied by side effects, such as obesity, insulin resistance, bone loss, hypertension, and dermal atrophy (25). Combination therapies employing glucocorticoids for adrenal replacement and androgen suppression (even 2 different glucocorticoids) as well as anti-androgens and androgen biosynthesis inhibitors for treatment of hyperandrogenism might be useful for treatment optimization and minimization of side effects. Treatment regimens and goals should be individualized, while these targets might be modified throughout patient's life. Laboratory data for adults with 21OH deficiency are useful as markers, but they are eventually less important than clinical evaluation. They can be improved by incorporating steroid profiling by mass spectrometry (26). When necessary, low doses of glucocorticoids may be used in patients with NCCAH (1). In these patients, when signs of hyperandrogenemia manifest, treatment is sometimes successful only with oral contraceptives alone or with spironolactone (1, 23).

Although most patients will become ovulatory with the routine dose of hydrocortisone, some will require greater doses for suppression of progesterone of adrenal origin. In patients who do not achieve pregnancy, progesterone plasma concentrations should be measured in the follicular phase of the menstrual cycle. In most cases adequate suppression of 17-hydroxyprogesterone results in adequate peripheral concentrations of adrenal-derived progesterone (although in this case one might not avoid exogenous hypercortisolism) (9, 20). Of note, greater doses of glucocorticoids are required when the therapeutic aim is the reduction of androgen concentrations, as compared to replacement doses required only for substitution of hormonal deficiency. For women who attempt to conceive when in glucocorticoid treatment, hydrocortisone, which is inactivated by the placenta, is employed. This treatment continues during pregnancy (1). Unilateral or bilateral adrenalectomy has been used as last resort for patients who do not respond to other treatments, especially those with “salt-wasting” CAH and large adrenal myelolipomas (most commonly developing in poorly controlled “classic” CAH) as well as in persistent hyperandrogenemia (3, 6), but it is not recommended because of its life-time invalidating risks (6, 8, 20, 27).

### Ovulation Induction in CAH

For those patients who cannot achieve ovulation despite adequate treatment and reduction of progesterone and androgen concentrations, gonadotropins or clomiphene may be useful to induce ovulation (20). *In vitro* fertilization (IVF) can be another treatment option for those, who fail to achieve pregnancy with these therapeutic means (8). By the time ovarian stimulation is achieved, the possibility to freeze all embryos and transfer them to a subsequent cycle should be considered in an effort to avoid the IVF protocol-induced increased progesterone concentrations. In cases where both parents are carriers of a CAH mutation or one parent is affected by CAH and the other is a carrier, there is an increased risk that the fetus will be affected from CAH. In this case it is essential to perform pre-implantation genetic diagnosis (PGD) (6, 8).

## Pregnancy in CAH Carriers and in CAH Patients

Prenatal diagnosis of CAH in the embryo or fetus can be done by performing chorionic villus sampling (9–11th week of pregnancy) or amniocentesis (15–20th week of pregnancy) followed by genetic testing (28). Specific probes for 21-hydroxylase mutations allow direct and rapid identification of known mutations through the use of polymerase chain reaction (i.e., allele specific). Panels of oligonucleotide probes, currently available for use in prenatal diagnosis, are expected to identify well more than 95% of current 21-hydroxylase mutations (4). In embryos belonging to a high-risk group for CAH, prenatal therapy to prevent virilization of external genitalia of a female embryo affected with CAH should be regarded as experimental. Recent studies address four areas of concern when dexamethasone is used as treatment: potential teratogenicity (cleft lip with/without cleft palate), reduced birth weight, potentially brain/behavior problems such as verbal working memory, reduced self-perception of scholastic competence and increased self-rated social anxiety, and potential long-term effects (insulin resistance) (27). There are no recommended specific treatment protocols and prenatal treatment should be obtained only through approved clinical protocols or trials (27). In embryos with increased probability of CAH (because of family risk), treatment with glucocorticoids should be introduced before the 9th week of pregnancy which effectively lowers excessive adrenal androgens amounts and thus, prevents masculinization of female external genitalia. The results from the villocentesis or amniocentesis will determine further patient's management. Treatment is discontinued when the fetus is male or unaffected female. Otherwise, it is continued until term in three divided doses based on maternal pre-pregnancy bodyweight (28). Concerns arise regarding the unnecessary corticoid treatment of pregnant women in case of male and unaffected female fetuses. Therefore, it is important to identify female affected fetuses before 9 weeks of pregnancy (4). Non-invasive techniques introduced in 2011 are based on extraction of fetal cell-free DNA (cfDNA) from maternal blood (28). This may become the new standard diagnostic approach in the future (4). The advantage of this test is that it can be done at the 6th week of pregnancy, allowing early diagnosis before the onset of genital organogenesis (9th week of pregnancy) and by that unnecessary treatments would be avoided (23, 28).

Spontaneous abortion rates appear to be greater, as compared to healthy pregnant women, in patients with CAH, as well as in patients with NCCAH who were not treated with glucocorticoids. These rates are normalized after glucocorticoid treatment (5, 8, 9). Pregnancies of women already diagnosed with CAH seem to be normal and uneventful (9). Genetic counseling is essential (5). Monitoring of pregnancy should be performed by a specialized team, which should include obstetrician, pediatrician and endocrinologist (5, 12). Symptoms of fatigue, nausea, and vomiting are common in pregnancy and overlap those of adrenal insufficiency. Overtreatment with hydrocortisone can lead to fluid retention, excessive weight gain and hypertension. Mothers should be evaluated for signs of adrenal insufficiency in pregnancy (i.e., postural hypotension) and stress dose steroids should be administered during labor. In the second and third trimester of pregnancy, the dose of hydrocortisone may need to be increased by 25–40%, although there is no consensus on this (5, 6, 8, 27). No dose adjustment of hydrocortisone is required in the early stages of pregnancy (9). Dose control of treatment with hydrocortisone during pregnancy should not be performed with plasma renin activity levels as they increase normally during pregnancy, but with testosterone and androstenedione concentrations (5, 6). Pregnant women with CAH appear to be at greater risk for developing gestational diabetes mellitus. The incidence of pre-eclampsia and premature delivery does not seem to change (5, 7, 9). Finally, cesarean section is preferable, especially for women who have prior genital reconstructive surgery, although vaginal deliveries have also been reported (5, 8, 9).

Babies from mothers with CAH and NCCAH have an increased risk to be small for gestational age (SGA) babies, especially when parents suffer from NCCAH. The long-term follow-up of the offspring has shown normal physical and intellectual development although these children might show deranged renal (particularly evident in females below the age of 5) and liver biochemistry (9, 29).

## Case Reports

In the literature there are a few cases of severe CAH which needed to undergo IVF. Albarel et al. reported a patient with StAR deficiency, homozygous for 1 bp delection in the StAR gene (719del). The patient, after missing ovarian response to clomiphene, underwent IVF with a long agonist protocol with 300 units menotropin per day. The procedure resulted in pregnancy with delivery of a normal female child (weight: 3.150 kg) at 40 weeks of gestation (30).

Bianchi et al. reported a 26 years old patient with CAH, associated with 17OH deficiency, a rare defect of steroid biosynthesis characterized by inability to synthesize cortisol, androgens or estrogens, complete absence of follicular maturation, hypergonadotropic hypogonadism, primary amenorrhea, and hypertension. The defect was due to a compound heterozygous mutation (p.W406R/P428L) in the *CYP17A1* gene. The patient underwent IVF with a long agonist protocol receiving 112.5 I.U. recombinant FSH per day. Four mature oocytes were retrieved and 3 blastocysts were obtained. Two of them were transferred and pregnancy was achieved. Pregnancy was complicated by pre-eclampsia, gestational diabetes (requiring insulin administration), cholestasis gravidarum (requiring ursacol administration), and cellulitis of the lower right extremity. At 30 weeks and 4 days, an emergency cesarean section was performed due to acute fetal distress. A true umbilical knot was identified, and a live normal male newborn was delivered (weight: 1,945 g; length: 43.5 cm) (31).

Neuwinger et al. also treated a 28 year female with 17OH deficiency. Because the ovaries of these patients contained numerous primordial follicles, the authors hypothesized that the absence of spontaneous follicular maturation could be due to a lack of aromatizable substrate. To provide this substrate, testosterone was administered either by intra-ovarian injection or by vaginal administration. Ovarian stimulation was performed with human urinary gonadotropins. Follicular maturation and ovulation were induced with this treatment, as confirmed by ultrasonography, measurement of LH, estradiol and progesterone serum concentrations and finally, aspiration of oocytes from the mature follicles. Fertilization of these oocytes *in vitro*, however, did not succeed (32).

Ben-Nun et al. reported the first viable pregnancy in a woman with 17OH deficiency in which embryos produced with donated oocytes were transferred to the uterus. At the fifth embryo transfer attempt, the treatment resulted in a twin pregnancy which was further complicated with severe pre-eclampsia, hemolysis, elevated liver enzymes, low platelet (HELLP) syndrome, and premature delivery. One newborn died minutes after delivery, whereas the other was kept for several weeks at the neonatal intensive care unit and discharged without apparent disabilities (33).

## Discussion

As previously described, the clinical presentation of “classic” CAH and NCCAH is in direct correlation with the genomic and biochemical background of the disease. Therefore, it is important to emphasize that treatment should be individualized. Moreover, it should be a matter of collaboration between health-providers of many disciplines.

Managing patients with “classic” CAH is challenging. These patients, in addition to treatment with glucocorticoids and mineralocorticoids depending on the form of the disease, might need surgical treatment of ambiguous genitalia. The right moment to operate these patients is a field of controversy. In the past, the decision on surgery was taken on the basis of appearance of external genitalia and the possibility of conception. However, in the past two decades it is preferred that surgery is postponed so that the patients gives his/her informed consent (1). Because of this controversy over gender behavior, gender identity, surgical outcome, and long-term sexual function, it is imperative to consider all therapeutic options on an individual basis (23).

In an infertility clinic, health professionals are much more frequently confronted with patients with NCCAH compared to patients with “classic” CAH because the former represent a larger undiagnosed population while the latter are identified early in infancy (20). Many patients with NCCAH are undiagnosed due to the mild symptoms that lead them to seek medical advice only in case of infertility (8). Moreover, there is a phenotypic overlap between NCCAH and PCOS (20). The distinction is made as women with NCCAH manifest greater concentrations of 17-hydroxyprogesterone and progesterone than women with PCOS, who present insulin resistance, obesity, polycystic ovary morphology, and increased LH/FSH ratios (28).

The main problems in NCCAH women are the increased progesterone concentrations which alter endometrial receptivity and tubal motility and lead to ovulation disorders. Appropriate therapy usually leads to regular menses and spontaneous pregnancies. Sometimes ovulation induction regimens (i.e., clomiphene) can be used as well as IVF techniques in case of insufficient ovarian function and pregnancy is not achieved.

## Conclusion

The treatment of infertility in CAH patients is a major challenge. Hydrocortisone is at the time being the gold standard treatment which restores ovarian function, ovulation, and endometrial receptivity. Performing PGD should be taken into consideration in cases where both parents are affected. Pregnancy should be followed by an expert team in a tertiary hospital in case of suspected affected fetus with CAH. Finally, patients with CAH should be followed by a multidisciplinary team including gynecologist, endocrinologist, and pediatrician.

## Author Contributions

AC and ES: literature review. RV and MP: manuscript. GM: revision.

### Conflict of Interest

The authors declare that the research was conducted in the absence of any commercial or financial relationships that could be construed as a potential conflict of interest. The reviewer GV declared a past co-authorship with one of the authors GM to the handling editor.
